# Development and Optimization of Cinnamon Oil Nanoemulgel for Enhancement of Solubility and Evaluation of Antibacterial, Antifungal and Analgesic Effects against Oral Microbiota

**DOI:** 10.3390/pharmaceutics13071008

**Published:** 2021-07-02

**Authors:** Khaled M. Hosny, Rasha A. Khallaf, Hani Z. Asfour, Waleed Y. Rizg, Nabil A. Alhakamy, Amal M. Sindi, Hala M. Alkhalidi, Walaa A. Abualsunun, Rana B. Bakhaidar, Alshaimaa M. Almehmady, Wesam H. Abdulaal, Muhammed A. Bakhrebah, Mohammed S. Alsuabeyl, Ahmed K. Kammoun, Adel F. Alghaith, Sultan Alshehri

**Affiliations:** 1 Department of Pharmaceutics, Faculty of Pharmacy, King Abdulaziz University, Jeddah 21589, Saudi Arabia; wrizq@kau.edu.sa (W.Y.R.); nalhakamy@kau.edu.sa (N.A.A.); wabuassonon@kau.edu.sa (W.A.A.); rbakhaidar@kau.edu.sa (R.B.B.); amnalmehmady@kau.edu.sa (A.M.A.); 2 Center of Excellence for Drug Research and Pharmaceutical Industries, King Abdulaziz University, Jeddah 21589, Saudi Arabia; 3 Advanced Drug Delivery Research Group, Faculty of Pharmacy, King Abdulaziz University, Jeddah 21589, Saudi Arabia; 4 Department of Pharmaceutics and Industrial Pharmacy, Faculty of Pharmacy, Beni-Suef University, Beni-Suef 62511, Egypt; rasha.mahmoud@pharm.bsu.edu.eg; 5 Department of Medical Microbiology and Parasitology, Faculty of Medicine, King Abdulaziz University, Jeddah 21589, Saudi Arabia; hasfour@kau.edu.sa; 6 Oral Diagnostic Sciences Department, Faculty of Dentistry, King Abdulaziz University, Jeddah 21589, Saudi Arabia; amsindi@kau.edu.sa; 7 Department of Clinical Pharmacy, Faculty of Pharmacy, King Abdulaziz University, Jeddah 21589, Saudi Arabia; halkhaldi@kau.edu.sa; 8 Department of Biochemistry, Faculty of Science, Cancer and Mutagenesis Unit, King Fahd Center for Medical Research, King Abdulaziz University, Jeddah 21589, Saudi Arabia; whabdulaal@kau.edu.sa; 9 Life Science and Environment Research Institute, King Abdulaziz City for Science and Technology (KACST), P.O. Box 6086, Riyadh 11442, Saudi Arabia; Mbakhrbh@kacst.edu.sa (M.A.B.); malsubyl@kacst.edu.sa (M.S.A.); 10 Department of Pharmaceutical Chemistry, Faculty of Pharmacy, King Abdulaziz University, P.O. Box 80260, Jeddah 21589, Saudi Arabia; akammoun@kau.edu.sa; 11 Department of Pharmaceutics, College of Pharmacy, King Saud University, P.O. Box 2457, Riyadh 11451, Saudi Arabia; afalghaith@ksu.edu.sa (A.F.A.); salshehri1@ksu.edu.sa (S.A.)

**Keywords:** cinnamon oil, nanoemulsion gel, optimization, oral health, pseudoplastic, surfactant

## Abstract

Oral health is a key contributor to a person’s overall health and well-being. Oral microbiota can pose a serious threat to oral health. Thus, the present study aimed to develop a cinnamon oil (CO)-loaded nanoemulsion gel (NEG1) to enhance the solubilization of oil within the oral cavity, which will enhance its antibacterial, antifungal, and analgesic actions against oral microbiota. For this purpose, the CO-loaded nanoemulsion (CO-NE) was optimized using I-optimal response surface design. A mixture of Pluracare L44 and PlurolOleique CC 497 was used as the surfactant and Capryol was used as the co-surfactant. The optimized CO-NE had a globule size of 92 ± 3 nm, stability index of 95% ± 2%, and a zone of inhibition of 23 ± 1.5 mm. This optimized CO-NE formulation was converted into NEG1 using 2.5% hydroxypropyl cellulose as the gelling agent. The rheological characterizations revealed that the NEG1 formulation exhibited pseudoplastic behavior. The in vitro release of eugenol (the marker molecule for CO) from NEG1 showed an enhanced release compared with that of pure CO. The ex vivo mucosal permeation was found to be highest for NEG1 compared to the aqueous dispersion of CO-NE and pure cinnamon oil. The latency reaction time during the hot-plate test in rats was highest (45 min) for the NEG1 sample at all-time points compared with those of the other tested formulations. The results showed that the CO-NEG formulation could be beneficial in enhancing the actions of CO against oral microbiota, as well as relieving pain and improving overall oral health.

## 1. Introduction

Overall health can be achieved only if the components leading to it are present. Oral health is one of the components that significantly contributes to the success of comprehensive healthcare [1]. The mouth is a host for several bacteria that are harmless when they are under control. However, the uncontrolled growth of these microbiota leads to the loss of oral health due to tooth and gum diseases. This can subsequently lead to other health issues of the cardiovascular and other systems [2].

The loss of oral health can be caused by bacteria, fungi, viruses, or protozoa [3]. Several thousands of species of oral microbiota exist, and they mainly occur as biofilms [4]. The presence of both core and variable microbiomes can be identified in the oral microbiome, and approximately 700 prokaryote species have been identified in it. Both Gram-positive and Gram-negative bacteria are present in a healthy oral cavity [5]. All of these species can be precursors of diseases of the oral cavity.

The effect of the oral microbiota on oral health depends on the microenvironment, signaling systems, host factors, and environmental factors. Imbalance in these factors can lead to dysbiosis and, ultimately, to the loss of oral health [6]. Most bacterial infections of the oral cavity are odontogenic. Dental caries, pulpitis, gingivitis, periodontitis, and pericoronitis are odontogenic. Syphilis, tuberculosis, leprosy, scarlet fever, and gonorrhea are some of the causes of nonodontogenic oral infections [7]. Most fungal infections are opportunistic in nature; oral candidiasis, caused by *Candida*, is the most commonly encountered oral fungal infection in clinical practice [8].

Essential oils, which are a rich source of mixtures of terpene hydrocarbons, have immense potential as natural remedies for oral infections. Lavender, eucalyptus, peppermint, clove, and cinnamon oils are the prominent essential oils for maintaining oral health [9]. The significant antimicrobial activities of these essential oils have been well demonstrated [10]. Cinnamon oil (CO), obtained from *Cinnamomum* plants of the Lauraceae family, has been well studied for its antibacterial activity and has been proven to be effective against both Gram-positive and Gram-negative organisms [11]. Its activity against *Streptococcus mutans* is specifically useful for the protection against dental cavities. Significant antifungal activity of CO has also been demonstrated. Interestingly, CO was found to be effective against fluconazole-resistant *Candida* species as well [12]. Cinnamaldehyde and eugenol are the major constituents of CO in 75% of formulations. Linalool, β-caryophyllene, eucalyptol, camphor, cinnamyl acetate, and eugenol are the other important constituents. Disruption of the cell membrane, facilitating the leakage of intracellular components by these compounds, has been demonstrated to be the mechanism underlying their antimicrobial action [13].

Essential oils trigger cell cycle arrest by means of the disruption of beta-tubulin distribution in *Candida* species. This subsequently leads to mitotic spindle defects and, thereafter, the leaking of intracellular components [14]. In addition to its antimicrobial actions, CO has established uses and applications in overall health and well-being. CO has anti-inflammatory activity. Further, it is a useful natural remedy against coughs, colds, constipation, and other disorders. CO facilitates digestion, stress reduction, and pain relief. It also stimulates the circulation of blood and lowers the blood pressure. CO can even be useful in controlling diabetes [15,16]. Because CO offers several advantages, the formulation of this essential oil in a suitable delivery system could be especially advantageous.

A nanoemulsion (NE) is an advanced delivery platform suitable for the enhancement of parameters such as solubility and stability [17]. Nanoemulsions consist of nanosized oil droplets stabilized by a mixture of surfactants and co-surfactants, and they are widely studied as drug delivery platforms [18]. The presence of globules of a nanometric size in the oil phase is the major reason behind the advantages of NEs. Therefore, the use of a CO nanoemulsion against oral microbiota is a promising approach [19].

Oral gel formulations situate the essential oil more effectively at the site of administration in the oral cavity. Therefore, conversion of a nanoemulsion to a gel formulation would be more appropriate [20]. Nanoemulsion gels (NEGs), prepared by the addition of a thickening or gelling agent to an NE, provide enhanced retention in the oral cavity and can even provide the sustained release of a medication [20,21]. Nevertheless, several factors are to be considered in the formulation, development, and optimization of NEs and NEGs. The design of experiments approach involves statistical methods and can be used for the optimization process in formulation development. Its use in the development and optimization of pharmaceutical formulations has been established [22,23]. Statistical optimization designs significantly reduce the number of trials and efforts needed to reach the optimum formula. Furthermore, the evaluation of the results is easy and can be conveniently extrapolated to several formulation variables.

The selection of the dose of any essential oil can pose some difficulties in formulation development. The reported minimum inhibitory concentrations of CO against *Escherichia coli*, *Staphylococcus aureus*, and *Pseudomonas aeruginosa* are 4.88, 4.88, and 19.53 μg/mL, respectively [24]. Interestingly, the MIC values of CO against biofilm causing *Staphylococcus epidermidis* were in the range of 0.5%–2% and these values were comparable or lower than chlorhexidine, triclosan, and gentamycin [25]. A study established a minimum CO concentration of 5% in NE formulations for antimicrobial activity against *Listeria* and *Salmonella* species [26]. The significant antimicrobial activities of CO led to the development of CO-based formulations. An anti-acne concealer with CO with activity against *Propionibacterium acnes* is one of these [27]. Core/sheath-structured nanofibers of CO have also been successfully tested for antimicrobial activity [28]. It is noteworthy that NEs are the most suitable formulation of essential oils such as CO [17].

Some NE formulations of CO have been reported for therapeutic applications. An NEG prepared with a blend of CO and usnic acid has been evaluated for its therapeutic potential in skin carcinogenicity [29]. However, the study did not evaluate the effect of cinnamon oil and HLB value on the NE formulation. Furthermore, it was tested for its action against skin carcinogenicity and not for oral application. Furthermore, the individual effect of CO was not evaluated. A similar disadvantage was noted with an NE using a blend of clove oil and CO [30]. Furthermore, there was no statistical optimization of the NE formulation in the study. However, a nanocream formulation was used to evaluate the effect of cinnamon leaf oil alone [31]. Unfortunately, the evaluation was limited to some in vitro and stability studies. Although the study assessed the effect of oil, S_mix_, and HLB value through the selection of some NE formulations using a pseudoternary diagram, no statistical evaluation or optimization of the NE formulation was carried out. In another study, CO was solubilized in coconut oil before nanoemulsification using different methods [32]. However, the study did not evaluate the individual effect of CO on the NE formulation. A more relevant study reported on the use of a CO-based NE against *Listeria* and *Salmonella* species [26]. However, the NE was intended for the melon industry and not for therapeutic applications. Moreover, no significant NE formulation was discussed in the study. Interestingly, a more recent study has reported the use of a CO NE against oral biofilms using this CO-based NE [33]. However, this study also omitted formulation aspects and focused on the antibacterial application alone.

Unfortunately, none of the above-mentioned reported studies were dedicated to the use of CO as the oil phase. Furthermore, the effect of a mixture of surfactants, instead of a single surfactant, has also not been tried for CO-based NEs. Moreover, the optimization of the NE formulation, considering hydrophilic-lipophilic balance (HLB) value as one of the constraints, has also not been tried for CO. Importantly, the formulation of an NEG suitable for an oral gel has also not been attempted for CO-based NEs. Thus, the present study was carried out to prepare and optimize CO as the oil phase of an NEG in order to enhance its antibacterial, antifungal, and analgesic actions and to improve oral health through the prevention of damage to the oral microbiota. The NE formulation, containing CO, was optimized and converted to an NEG. The CO-loaded NEG (CO-NEG) formulation was further evaluated.

## 2. Materials and Methods

### 2.1. Materials

Cinnamon oil was obtained as a gift sample from the Verywell Health Company (New York, NY, USA). Pluracare L44 (HLB = 16) was purchased from the BASF SE Chemicals Company (Ludwigshafen, Germany). PlurolOleique CC 497 (HLB = 3) and Capryol PGMC were obtained as gift samples from Gattefosse (Chemin de Genas 69800 Saint Priest, France). Hydroxypropyl cellulose, methanol, acetonitrile, phosphate buffer (HPLC grades) were purchased from Sigma Aldrich (St. Louis, MO, USA).

### 2.2. Construction of Pseudoternary Phase Diagram

Cinnamon oil, surfactant (Pluracare L44 to PlurolOleique CC 497 in a ratio of 1:1), and co-surfactant (Capryol PGMC) were used to prepare the NE. The minimum CO concentration of 5% in NE formulations for antimicrobial activity has been already identified against some species [26]. Therefore, in this study, we aimed to obtained the highest possible range of CO concentrations above 5% for the optimization. The selection of surfactants and co-surfactants also has an immense role in the success of the NE formulation. Towards this objective, a combination of surfactants was planned instead of a single surfactant. Pluracare L44, a nonionic surfactant of poloxamer class, was one of the surfactants selected, and it has the ability to form both o/w and w/o emulsions. PlurolOleique CC 497 is a water-insoluble surfactant that helps in nanoemulsification. PlurolOleique CC 497 is particularly useful for the solubilization of poorly soluble drugs and thus is a good choice for the nanoemulsification of CO. Thus, a combination of Pluracare L44 and PlurolOleique CC 497 can be expected to provide the maximum benefit of each and result in a robust and stable NE formulation of CO. Capryol PGMC, chemically propylene glycol monocaprylate, is a solubilizer and surfactant for nanoemulsion formulations. It is suitable for lipid-based formulations and it was expected that its use as co-surfactant in CO-based NE would be appropriate. The surfactant and co-surfactant were mixed at different mass ratios (1:1, 2:1, 3:1, 4:1, 1:2, 1:3, and 1:4) to prepare the S_mix_. Different combinations of oil and S_mix_ were made so that the maximum ratios were covered in order to precisely delineate the boundaries of the phases formed in the phase diagrams. The pseudo-ternary-phase diagrams of the oil, S_mix_, and water were developed using the aqueous titration method. Various combinations were identified to study the NE region in the generated pseudoternary-phase diagram.

### 2.3. Formulation and Optimization of CO Nanoemulsion (CO-NE)

#### 2.3.1. Experimental Design

The I-optimal response surface experimental design was selected for the optimization of the CO-NE formulation. Based on the ternary phase diagram, the levels for the three independent variables were selected. The CO (Factor A) was studied at 12%, 16%, and 20% in the CO-NE formulation. The S_mix_ percentage was the second factor (Factor B) selected and was studied at 30%, 35%, and 40%. Furthermore, the HLB value of the mixture of surfactants (Factor C), Pluracare L44 and PlurolOleique CC 497, was studied at 10, 12, and 14. The rationale for selecting those independent variables was the aim of the study, which was the formulation of a cinnamon oil nanoemulsion; the CO is the active constituent in the formulation, so it is essential to incorporate within the formula. During the formulation of the nanoemulsion, certain percentages of the surfactant and cosurfactant mixture (S_mix_) are usually required to divide the oil globules within the nanosize range and produce the nanoemulsion, so the S_mix_ percentage was also selected as an independent variable. Furthermore, each oil has its own required HLB to be divided into fine dispersed droplets within the continuous phase of the emulsion, so the value of HLB also selected as one of the independent factors. The selected dependent variables were also selected according to the aim of the research, which was the formulation of a cinnamon oil nanoemulsion, so the measurement of the droplet size was selected as one of dependent variables. In addition, to ensure the stability of this nanoemulsion—which is one of the dispersed dosage forms which faces stability problems compared to solid dosage forms such as tablets—the stability index was also chosen as one of the factors. The third factor chosen was the inhibition zone against *Streptococcus mutans*, as the major aim of the study was the improvement of oral health against oral microbiota, and *Streptococcus mutans* is one of the microorganism that exerts major effects on oral health. The globule size, stability index, and zone of inhibition (a measure of antibacterial activity) against *Streptococcus mutans* were taken as the responses for evaluation and optimization. Twenty-one runs (formulations) were prepared randomly by the simple mixing of selected components. The total of three components (CO, S_mix_, and water) in the mixture was kept equal to 100%.

#### 2.3.2. Evaluation of NE Formulations

##### Droplet Size

Approximately 500 µL of the NE formulation was diluted with distilled water (1:10 *v*/*v*) and analyzed for globule size by means of dynamic light scattering (Zetatrac, Microtrac, Montgomeryville, PA, USA).

##### Stability Index

The NE formulations were exposed to temperature changes to assess their thermodynamic stability. The NE formulations, which had previously been assessed for globule size, underwent three freeze–thaw cycles consecutively, with freezing at −25 °C and thawing at 25 °C, both for 12 h. After completion of the three cycles, the resultant sample was tested once again for globule size. The stability index was determined using the initial and final globule sizes, as shown in Equation (1).

(1) $$\mathrm{Stability}\;\mathrm{index}\;=\frac{\mathrm{Original}\;\mathrm{globule}\;\mathrm{size}\;-\;\mathrm{Change}\;\mathrm{in}\;\mathrm{globule}\;\mathrm{size}}{\mathrm{Original}\;\mathrm{globule}\;\mathrm{size}}\times 100\;$$

##### Zone of Inhibition by CO-NE

To evaluate the antimicrobial activity in terms of the zone of inhibition, the *S. mutans* strain (ATCC 25175, Microbiologics, St. Cloud, MN, USA) was selected as the representative microorganism. The disk diffusion method was used for the determination of the zone of inhibition of the samples. Filter paper discs (of 6 mm in diameter) placed in a Petri dish were impregnated with 50 µL of each of the experimental NE formulations after sterilization in a hot-air oven (160 °C for 2 h). Four disks per plate were used in the study and incubated for 24 h at 37 °C to allow for the formation of inhibition zones around the disks. The zone of inhibition was determined using a caliper in triplicate and reported.

#### 2.3.3. Optimization of CO-NE Formulation

The optimization of the CO-NE formulation was done as per the constraints mentioned in Table 1. The optimization was carried out using the CO percentage (Factor A), S_mix_ percentage (%) (Factor B), and HLB value (Factor C) with “in range” specifications. The minimum value for the droplet size and the maximum values for the stability index and zone of inhibition were chosen for the response variables.

### 2.4. Preparation of the Optimized CO-NEG Formulation

The optimized CO-NE formulation was converted to an NEG using hydroxypropyl cellulose (HPC) as the gelling agent. Briefly, 2.5% HPC was sprinkled over 100 mL of CONE prepared via the dilution of 25 mL of CO-NE with 75 mL of distilled water and the dispersions were stirred at 500 rpm until the polymeric chains of HPC were homogeneously dissolved. The mixture was then sonicated in a bath sonicator for 3 min to ensure dissolution and prevent any clumping of the HPC. Afterward, the CO-NEG was stored in the refrigerator for 24 h before further characterization in order to remove any entrapped air bubbles. Different CO-loaded HPC hydrogels, as shown in Table 2, were prepared in the same manner.

### 2.5. Rheological Evaluation of the HPC Hydrogel Loaded with Optimized CO-NE (NEG1)

The rheological characterization of NEG1 was compared with plain HPC hydrogel (G4). The sample (1 g) was subjected to characterization at 25 °C ± 1 °C using a Brookfield viscometer with spindle 52. The shear rates of 2, 10, 20, 30, 40, 50, and 60 s^−1^ were employed in the study. The flow curves were plotted for the samples. The Farrow’s constant (n) was determined using Equation (2).

(2) Log G =n Log F –Log ɳ

where G is the shear rate, ղ is the viscosity, F is the shear stress, and n is the Farrow’s constant.

### 2.6. In Vitro Release of CO from HPC Hydrogel Loaded with Optimized CO-NE (NEG1)

Eugenol, a major chemical constituent of CO, was chosen as the marker molecule to study the in vitro release of CO from the NEG1. The study compared the release of eugenol from NEG1 with its release from G3 and pure CO using a USP Type I (basket) dissolution apparatus with modifications. Instead of the basket, cylindrical tubes (10 cm in length and 2.7 cm in diameter) were used. The samples were placed in the tubes after securing the lower part of the tubes with a semipermeable membrane (10 µm in pore size). The study was carried out at 50 rpm using 250 mL of phosphate buffered saline at a pH of 6.8 as the dissolution medium at 37 °C ± 0.5 °C for 2 h. The samples were collected, filtered (0.45-µm membrane filter), and analyzed via high-performance liquid chromatography. The analysis of injected samples (20 μL) was carried out with methanol and water in a volume ratio of 75:25 run at 1 mL/min in a C18 Phenomenex column (250 mm × 4.6 mm, 5 μm) and detected at 280 nm, employing a UV/Vis detector.

### 2.7. Ex Vivo Mucosal Permeation Studies

As in the in vitro release studies, eugenol was used as the marker molecule for assessing the ex vivo skin permeation of CO from NEG in comparison with G3 and CO. Briefly, fresh sheep buccal mucosa (2 cm × 2 cm and a diffusion area of 1.75 cm^2^) was placed in the Franz diffusion cell with phosphate buffered saline (pH 6.8) as the receptor fluid and maintained at 37 °C ± 1 °C and 400 to 420 rpm. The mucosal permeation of eugenol from NEG1, G3, and pure CO was estimated. Samples were withdrawn and analyzed for eugenol content using the high-performance liquid chromatography method described in the in vitro release study.

### 2.8. In Vivo Analgesic Activity on Rats

After approval of the protocol by the local Institutional Review Board for Preclinical and Clinical Research (Approval No. 14-4-2021), the in vivo studies were performed in four groups (six animals in each group) of adult male Wistar rats (200 to 300 g), with one group each for NEG1, NEG2, G3, and G4. The pain reaction time recorded pretreatment was taken as the basal threshold. The samples were then applied to the fore and hind limbs. The onset and duration of the analgesic effect of the NEG1, NEG2, G3, and G4 samples were tested by the hot-plate method at 55 °C ± 1 °C. The latency reaction time was recorded after 5, 15, 30, 45, and 60 min. The prolongation of the latency times were compared for the samples, and the results were expressed as the mean ± SD.

## 3. Results and Discussion

### 3.1. Construction of Pseudoternary Phase Diagram

**Figure 1:**
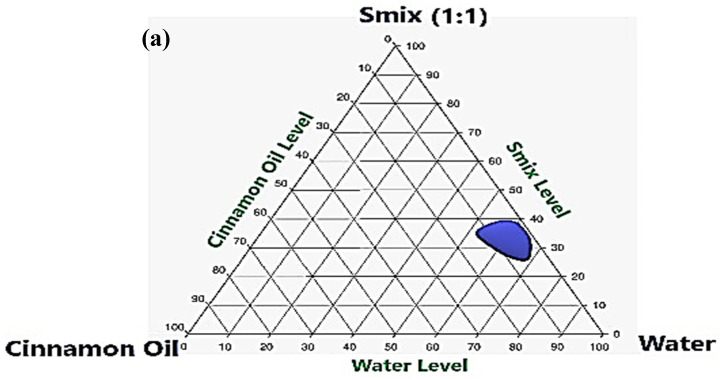

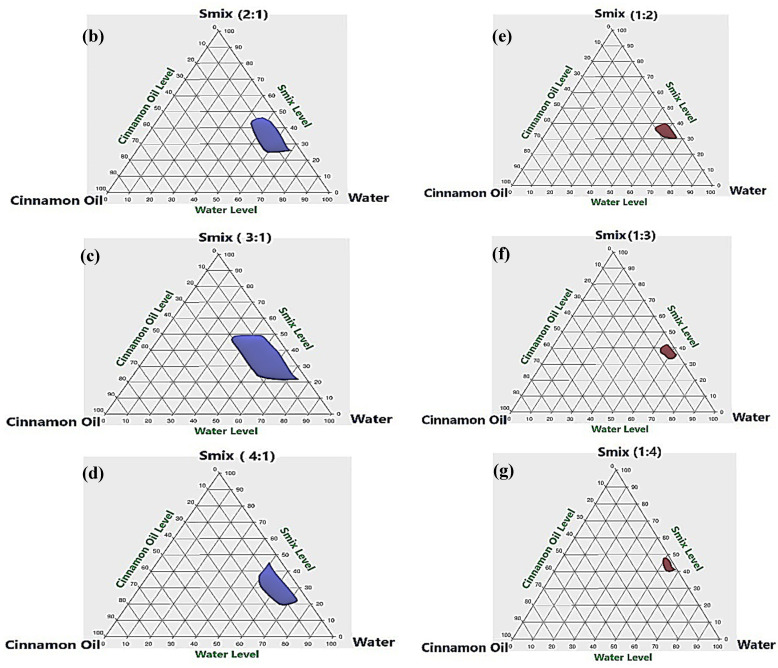
Pseudoternary-phase diagram constructed for the identification of the nanoemulsion region using cinnamon oil (CO), surfactant (Pluracare L44 and Plurol Oleique CC 497 in a ratio of 1:1), and co-surfactant (Capryol PGMC) at different surfactant and co-surfactant mixture (S_mix_) ratios: (**a**) S_mix_ 1:1, (**b**) S_mix_ 2:1, (**c**) S_mix_ 3:1, (**d**) S_mix_ 4:1, (**e**) S_mix_ 1:2, (**f**) S_mix_ 1:3, and (**g**) S_mix_ 1:4.

Seven ternary phase systems were constructed (Figure 1) using S_mix_ prepared at a mass ratio of 1:1, 2:1, 3:1, 4:1, 1:2, 1:3, and 1:4. In the S_mix_ of 1:1, the maximum concentration of CO that could be solubilized was 12% using 36% S_mix_ and 52% water. As the surfactant concentration was increased in the S_mix_ (2:1, 3:1, and 4:1), larger NE regions were observed. At an S_mix_ ratio of 2:1, 16% CO was emulsified using 30% S_mix_ and 54% water. By increasing the surfactant level in S_mix_ (ratio 3:1), a greater increase in the NE region was observed and 21% CO was emulsified using 40% S_mix_ and 39% water. However, a further increase in the surfactant level in S_mix_ (ratio 4:1) led to a decrease in the NE region compared with that obtained for an S_mix_ ratio of 3:1, and only 15% CO was emulsified using 30% S_mix_ and 55% water. When the concentration of Capryol PGMC co-surfactant was increased with respect to the surfactant mixture (1:2, 1:3, and 1:4), the NE area was decreased. The quantities of CO that could emulsify were 11%, 8%, and 5%, with S_mix_ ratios of 1:2, 1:3, and 1:4, respectively. Hence, the S_mix_ ratio of 3:1 was used in the rest of the experimental design.

### 3.2. Formulation and Evaluation of CO Nanoemulsion (CO-NE)

#### 3.2.1. Experimental Design

The results of the formulation trials are shown in Table 3. The droplet size, stability index, and zone of inhibition were determined and reported. The predicted values of the responses are also provided.

##### Droplet Size

A two-factor interaction (2FI) model was suggested for the analysis of the globule size of CO-NE. The analysis of variance (ANOVA) data for the globule size of CO-NE indicated that the model was found to be significant, and the factors B, C, and BC were found to have a statistically significant influence on the globule size of the CO-NE (Supplementary Table S1). The lack of fit was significant. Nevertheless, the predicted R-squared (0.9796) and adjusted R-squared (0.9901) values were in good agreement. Furthermore, there was a good correlation between the observed and predicted values of the droplet size (Table 3).

**Figure 2:**
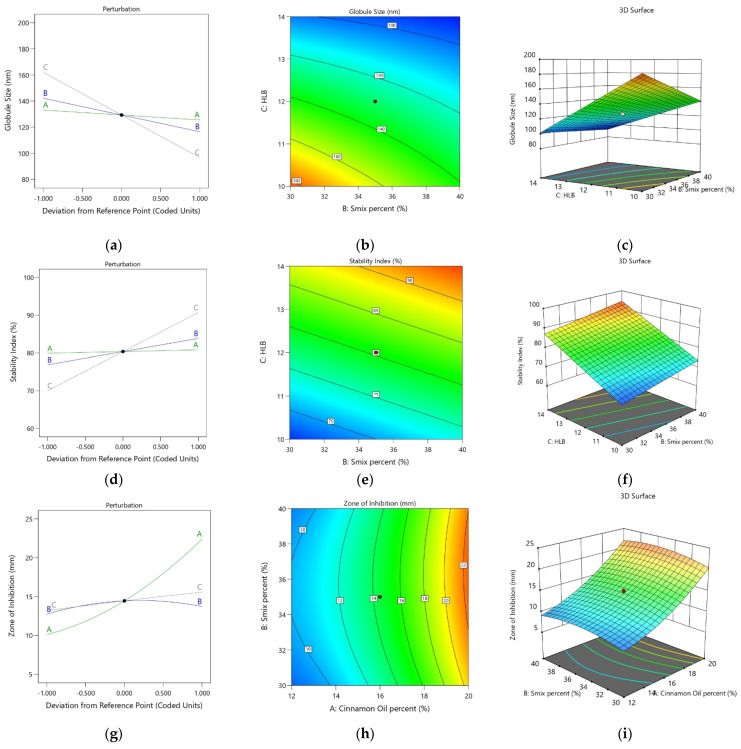
Statistical design plots for the droplet size, stability index, and zone of inhibition of CO-NE: (**a**) perturbation plot for globule size, (**b**) contour plot for droplet size,(**c**) response surface plot for droplet size, (**d**) perturbation plot for stability index, (**e**) contour plot for stability index, (**f**) response surface plot for stability index, (**g**) perturbation plot for the zone of inhibition, (**h**) contour plot for the zone of inhibition, and (**i**) response surface plot for the zone of inhibition.

The equation for the globule size derived in terms of the coded factors is provided in Equation (3) The plots resulting from the analysis of the droplet size of the CO-NE formulations are shown in Figure 2. The perturbation plot (Figure 2a) showing the individual effects of the factors confirmed that the HLB value (Factor C) had the greatest effect on the droplet size. Meanwhile, the CO concentration (Factor A) had the least influence. Increasing the HLB value was found to decrease the droplet size. In general, higher HLB values are favorable for o/w-type nanoemulsions. Most studies report an optimum HLB value depending on the required HLB of the oil phase for the preparation of stable NEs [34]. Nevertheless, higher HLB values indicate better hydrophilicity of the surfactants and the possibility of the formation of an o/w emulsion with a smaller droplet size. Furthermore, it has already been demonstrated that the particle size of nanoparticles tends to be small at higher HLB values [35]. Meanwhile, a higher S_mix_ concentration (Factor B) led to a smaller droplet size. Surfactants and co-surfactants can decrease the interfacial tension between the CO and aqueous phase and subsequently decrease the energy required to form the nanosized CO droplets, and this finally results in a lessening of the droplet size. In addition, the presence of Pluracare^®^ L 64 (poloxamer 184), a hydrophilic polymer, in the mixture of surfactants could form a protective coating around the nanodroplets, preventing their coalescence and enhancing their stability [36]. In general, the droplet size increases with an increase in the oil concentration. However, only a minuscule effect of CO (Factor A) was observed on the globule size, and, surprisingly, the droplet size was reduced rather than increased as expected. Interestingly, terpenes are the major constituents of CO, and these compounds have shown an in vitro lowering of the surface tension [37]. Therefore, this surface tension-lowering effect of terpenes in CO might have contributed to this slightly unanticipated effect. The contour plot (Figure 2b) and response surface plot (Figure 2c) show the overall response of the droplet size. Based on these plots, it was confirmed that a lower surfactant concentration increases the globule size, whereas higher HLB values favor a small droplet size.

Droplet size = + 129.36 − 3.78 A − 12.83 B − 32.67 C + 1.62 AB + 1.07 AC + 7.90 BC(3)

##### Stability Index

In the case of the stability index, a linear model was suggested by the design. The ANOVA data for the stability index of CO-NE indicated that the design model was found to be significant with a nonsignificant lack of fit. The ANOVA data revealed that Factors B and C were found to have a statistically significant influence on the stability index of CO-NE (Supplementary Table S2). Furthermore, the predicted R-squared and adjusted R-squared (0.9901) values (0.9567 and 0.9661, respectively) matched well with each other. Furthermore, a good correlation was shown for the observed and predicted values of the stability index (Table 3).

The polynomial equation, in terms of coded factors suggested by the software for the stability index of the CO-NE formulations, is provided in Equation (4). The linear equation confirmed that the effect of CO was not significant compared with the S_mix_ percentage and HLB value. The positive sign for the coefficients of Factors B and C indicated that both the S_mix_ percentage and the HLB value increased the stability index of the CO-NE. This result was further confirmed by the perturbation plot for the stability index (Figure 2d). In addition, the highest effect of Factor C (HLB value) was observed in the perturbation plot. A high value of the stability index indicated a good stability of the CO-NE formulation for resisting the changes in globule size. Thus, the factors that provided greater stability of the nanoemulsion globules could be expected to increase the stability index. Because higher HLB values indicated higher hydrophilicity, the stability of the o/w-type CO-NE could be enhanced at higher values of HLB. An increase in the S_mix_ percentage was also found to increase the stability index. The capability of the surfactant and co-surfactant to reduce the interfacial tension and provide better stability for the CO-NE droplets was the most prominent reason for such an observation. Furthermore, the presence of Pluracare^®^ L 64 (poloxamer 184) in the mixture of surfactants could provide a protective coating to the globules, thereby significantly enhancing the droplet stability [36]. The effect of the CO concentration on the stability index was statistically not significant. These observations were further confirmed by the two-dimensional (contour) and three-dimensional (response surface) plots provided by the software. In the contour plot (Figure 2e), the iso-value curves were found to significantly change as the HLB value changed, confirming its significant effect on the stability index. Meanwhile, the elevation of the response surface (Figure 2f) was observed more toward the highest values of the S_mix_ percentage and highest HLB values, confirming the increase in the stability index of the CO-NE with an increasing S_mix_ percentage and HLB value.

Stability index = + 80.36 + 0.4557 A + 3.49 B + 10.35 C (4)

##### Zone of Inhibition

Unlike the results for globule size and stability index, a quadratic model was suggested for the zone of inhibition. The ANOVA data for the zone of inhibition of CO-NE indicated that the selected model was significant and factor A has a significant effect on the zone of inhibition of CO-NE (Supplementary Table S3). The predicted R-squared value was 0.8872 and the adjusted R-squared value was 0.9757, showing reasonable agreement. Moreover, the observed and predicted values were also in agreement (Table 3).

The equation for the zone of inhibition suggested by the design based on the experiment trials is shown in Equation (5). The ANOVA data, polynomial equation, and perturbation plot (Figure 2g) confirmed the highest and significant effect of the CO concentration (Factor A) on the zone of inhibition. A drastic increase in the zone of inhibition was observed with an increase in the percentage of CO in the CO-NE. The significant antimicrobial activity of CO against *S. mutans* can be explained as the reason for this observation [38]. Meanwhile, the S_mix_ percentage did not show any statistically significant effect on the zone of inhibition. A minuscule effect of the HLB value on the zone of inhibition was observed. It is known that surfactants have antimicrobial activity and can enhance the activity of other antimicrobial agents [39]. It is also reported that Tween 80, a surfactant with a high HLB value, significantly enhances the antimicrobial activity of CO [32]. The HLB value in the present study was modified with the use of a mixture of Pluracare L44 and Plurol Oleique CC 497. The HLB value of poloxamers, such as Pluracare L44, is high, and the proportion of Pluracare L44 in the mixture of surfactants will be greater at higher HLB values. Therefore, this might have also contributed to the increase in the zone of inhibition at higher HLB values. Figure 2h,i show the contour and response surface plots, respectively, for the zone of inhibition. The iso-value curves in the contour plot are nearly perpendicular to the axis of CO, showing its significant effect on the zone of inhibition. Similarly, a significant change in the elevation of the response surface along the axis of the CO was observed. Meanwhile, no significant change in the elevation of the response surface was observed along the axis of the S_mix_ percentage.

Zone of inhibition = + 14.46 + 6.16 A + 0.4880 B + 1.19 C + 0.2609 AB + 0.5542 AC − 0.3839 BC + 1.82 A² − 1.22B² − 0.0738 C²(5)

#### 3.2.2. Optimization of CO-NE Formulation

The optimum CO-NE formulation with 20% CO, 40% S_mix_, and an HLB value of 14 (Table 4) was suggested by the design. The design suggested several optimized formulations, but the selection depended on the target goal for every response, which was the minimization of particle size, the maximization of the stability index, and the maximization of the zoon of inhibition. (Supplementary Table S4) The optimized formula (CO-NE) prepared was evaluated for its droplet size, stability index, and zone of inhibition. The observed droplet size, stability index, and zone of inhibition were found to be 92 ± 3 nm, 95 ± 2%, and 23 ± 1.5 mm, respectively. The observed responses were found to be close to the predicted ones.

### 3.3. Rheological Evaluation of the HPC Hydrogel Loaded with Optimized CO-NE (NEG1)

**Figure 3:**
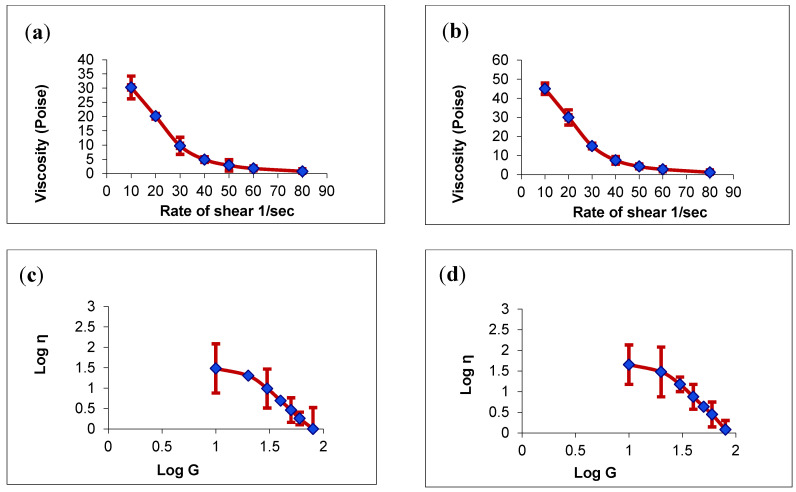
Plots of the shear rate (G) versus the viscosity (η) with a normal scale and with a logarithmic scale. (**a**,**c**) for plain hydroxypropyl cellulose (HPC) hydrogel (G4) and (**b**,**d**) for HPC hydrogel loaded with optimized CO-NE (NEG1). Values are expressed as the mean ± SD (*n* = 3).

The pseudoplastic or shear-thinning properties of the gel samples were observed in the rheological characterization (Figure 3). These observations were in good agreement with previous reports on the pseudoplastic behavior of HPC hydrogels [40]. The HPC concentration in the hydrogels was 2.5%, and this value was significantly higher than the threshold value of 1.5% reported to produce pseudoplastic flow. Furthermore, it was seen that both the ɳ_min_ and ɳ_max_ were higher for the NEG1 sample. The presence of Pluracare^®^ L 64 (poloxamer 184), an excellent thickening agent, as a component of the surfactant mixture, probably led to the higher viscosity of the NEG1 sample. Furthermore, hydrogel viscosity is subject to enhancement when lipid-based nanostructures are included in it [41]; this might also have contributed to the higher viscosity of NEG1.

**Figure 4:**
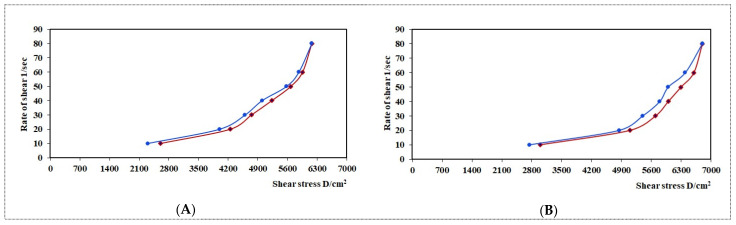
Rheograms of (**A**) plain hydroxypropyl cellulose (HPC) hydrogel (G4) and (**B**) HPC hydrogel loaded with optimized CO-NE (NEG1).

Figure 4 shows the rheograms of the rate of shear versus shear stress observed for the hydrogel samples. The rheogram of the plain HPC hydrogel (G4) shows very insignificant hysteresis in the rheogram. This behavior—the negligible hysteresis of HPC hydrogels—has already been reported [40]. Meanwhile, the hysteresis area was found to be slightly increased in the NEG1 sample. Because the hysteresis area is a measure of thixotropic behavior, it can be concluded that the NEG1 sample has enhanced thixotropic behavior compared with plain HPC hydrogel. The presence of Pluracare^®^ L 64 (poloxamer 184) probably led to this behavior. This argument is supported by the results of a previous study showing that poloxamer enhances the thixotropic behavior of lactose/poloxamer dispersions [42]. The enhancement of thixotropy in NEG1 provides the additional advantage of easy application and the enhanced retention of the gel formulation in the oral cavity.

**Figure 5:**
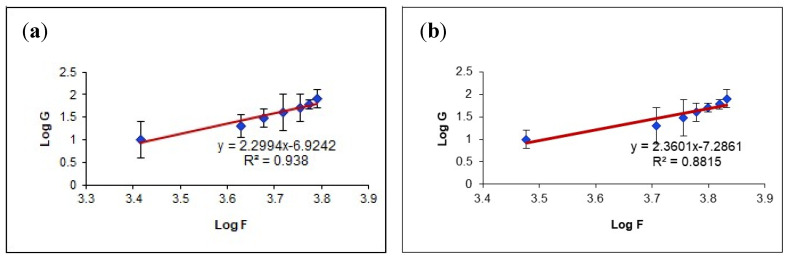
Plots of the logarithm of the shear rate (G) versus the logarithm of the shear stress (F) for (**a**) plain HPC hydrogel (G4) and (**b**) HPC hydrogel loaded with optimized CO-NE (NEG1). Values are expressed as the mean ± SD (*n* = 3).

A plot showing the log shear rate versus the log shear stress was obtained for both samples (Figure 5). Farrow’s constant, the slope of these plots, was determined for both samples. A Farrow’s constant value greater than one confirms a shear-thinning system [43]. Thus, it was found that both samples had Farrow’s constant values greater than one. The Farrow’s constant values were 2.2994 and 2.3601 for the G4 and NEG1 samples, respectively. Thus, only a slight increase in pseudoplastic behavior can be expected from the NEG1 sample.

### 3.4. In Vitro Release of CO from HPC Hydrogel Loaded with Optimized CO-NE (NEG1)

**Figure 6:**
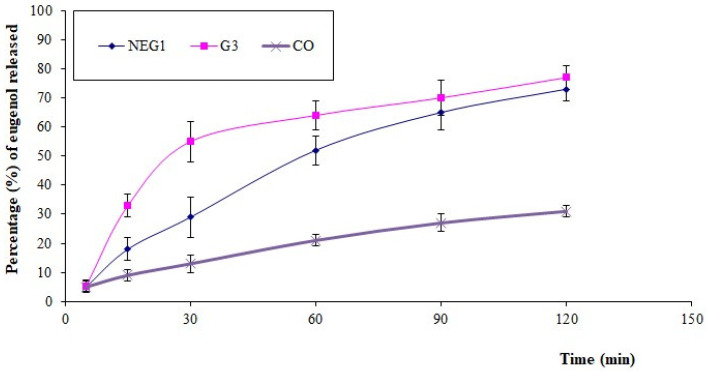
In vitro eugenol (marker molecule for CO) release from HPC hydrogel loaded with optimized CO-NE (NEG1), aqueous dispersion of CO-NE without HPC gel base (G3), and pure cinnamon oil (CO).

The in vitro release of eugenol (marker molecule for CO) from NEG1 was studied in comparison with the G3 and CO samples. The results (Figure 6) showed a significant increase in dissolution compared with pure CO. This significant increase in the percentage of eugenol from the NE formulation could be due to the presence of oil in the nanosize range within the formulation, and the presence of surfactants and cosurfactants within the NE may have enhanced the solubilization of oil within the dissolution media. A nanoemulsion formulation has already been reported to enhance the solubility and dissolution of essential oils due to the presence of surfactants and co-surfactants and a small droplet size [17]. Moreover, pure CO is poorly soluble and immiscible with the dissolution medium. Therefore, the observation of the very high dissolution and release of eugenol can be justified. Meanwhile, a sustained release of eugenol was observed from NEG1 in comparison with the G3 sample. This sustaining in the release of eugenol from NEG1 could be due to the viscosity of the HPC gel base, which could retard the release compared with the aqueous dispersion of NE, which enhanced the solubility and dissolution of the essential oil but could not sustain its release. Obviously, the conversion of an NE to an NEG using a gelling agent provided a diffusion barrier for the drug release, leading to a sustained or controlled drug release [44]. Such an effect could be the reason for the sustained release of eugenol (and, thus, CO) from NEG1.

### 3.5. Ex Vivo Mucosal Permeation Studies

**Figure 7:**
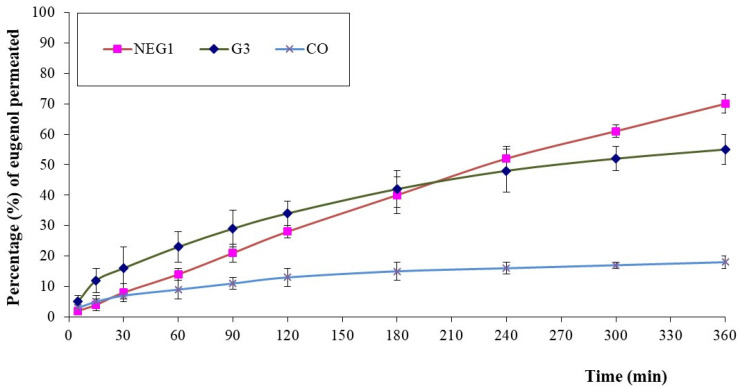
Results of ex vivo skin permeation of eugenol (marker molecule for CO) from HPC hydrogel loaded with optimized CO-NE (NEG1), aqueous dispersion of CO-NE without HPC gel base (G3), and pure cinnamon oil (CO).

The results of the ex vivo permeation study of eugenol (marker molecule for CO) from NEG1 (Figure 7) showed a pattern similar to that observed in the in vitro release study. In comparison with CO, the permeation was significantly high with NEG1. Essential oils are established permeation enhancers due to the rich presence of terpenes. Nevertheless, the immiscibility of these essential oils in an aqueous phase poses problems in mucosal permeation when they are used alone, and therefore a vehicle that can improve its wettability by reducing the interfacial tension is desirable. NE and NEG formulations provide such an advantage and show enhanced permeation. When comparing NEG1 with G3, the initial permeation was greater with the G3 sample, but at later time points, it was greater with NEG1. NEs improve the solubility and permeability of essential oils [17]. Furthermore, because G3 is a simple aqueous dispersion of CO-NE without HPC, the release of CO from G3 is faster compared with NEG1, which is a hydrogel. Therefore, the initially higher ex vivo permeation could be due to a greater drug release from the G3 sample. This argument could be justified by the enhanced release of eugenol observed during the in vitro release study. At later time points, the hydrogel NEG1 sample could provide a sustained release of CO, making it available for permeation through the mucosal membrane. However, such a continuous supply of CO from a G3 sample for permeation is not possible. Thus, the net result will be an increased mucosal permeation of CO from NEG1 at later time points. The results of the study were in good correlation with this mechanism.

### 3.6. In Vivo Analgesic Activity on Rats

The results of the in vivo study confirmed the significant anesthetic action of NEG1 in terms of the latency reaction time in rats (Table 5). NEG1 and G3 produced latency reaction times of 18.5 ± 1.5 and 14.5 ± 1.0 s, respectively, after 5 min. These were significantly higher (*p*-value < 0.05) than those observed for the NEG2 (9.5 ± 1.5 s) and control (8.0 ± 0.5 s) samples after the same 5-min time interval. Similarly significant (*p*-value < 0.05) effects of the NEG1 and G3 samples, compared with the controls, were observed after 15 and 30 min. After 45 min, the effect of G3 was significantly reduced. The effect of NEG1 was significantly high (*p*-value < 0.05) even after 45 min. The effect of NEG1 reached the baseline after 60 min. Thus, the duration of analgesia was 30 and 45 min for the G3 and NEG1 samples, respectively. All these observations indicated that the CO-loaded NE has more significant anesthetic activity than the G4 control gel. Furthermore, the formulation of the NEG of the CO-NE enhances the latency reaction time and thereby prolongs its anesthetic activity. Moreover, the use of CO (in NEG1) instead of oleic acid (in NEG2) significantly increases the anesthetic activity. Moreover, the significant local anesthetic activity demonstrated by NEG1 in rats compared with these results could be justified on the basis of the established analgesic activity of CO [45].

## 4. Conclusions

The S_mix_ for the formulation of CO-NE was prepared with a mixture of Pluracare L44 and Plurol Oleique CC 497, in a ratio of 1:1, as the surfactant and Capryol PGMC as the co-surfactant. The pseudoternary phase diagrams revealed that the S_mix_ (surfactant-to-co-surfactant) ratio of 3:1 provided the largest area for the nanoemulsion region. Subsequently, the CO-NE formulation was optimized using an I-optimal response surface design with a CO percentage, the S_mix_ percentage, and the HLB value of the mixture of Pluracare L44 and Plurol Oleique CC 497 as independent factors. The globule size, stability index, and zone of inhibition were taken as the responses for the optimization. The optimum CO-NE formulation contained 20% CO and 40% S_mix_ and had an HLB value of 14 for the mixture of surfactants. The optimized CO-NE had a globule size of 92 ± 3 nm, a stability index of 95% ± 2%, and a zone of inhibition of 23 ± 1.5 mm. The CO-NE formulated was converted to CO-NEG using 2.5% HPC and was further evaluated. The rheological characterization revealed the pseudoplastic behavior of CO-NEG. The in vitro release of eugenol (a marker molecule for CO) from NEG1 showed an enhanced release and a sustained release profile compared with the CO and G3 samples, respectively. The ex vivo mucosal permeation confirmed the enhanced permeation of eugenol from NEG1. Meanwhile, the NEG1 formulation was found to increase the latency reaction time to 45 min. Thus, the results of the present study confirmed that a formulation of CO in an NEG could be beneficial in order to combat the adverse actions of oral microbiota.

## Figures and Tables

**Table 1:** Constraints selected for the optimization of the CO-NE formulation.

Factors and Responses		Goal	Lower Limit	Upper Limit
Factor A	Cinnamon oil percent	is in range	12%	20%
Factor B	S_mix_ percent	is in range	30%	40%
Factor C	HLB value	is in range	10	14
Response 1	Droplet size	minimize	89 nm	191 nm
Response 2	Stability index	maximize	65%	96%
Response 3	Zone of inhibition	maximize	7 mm	24 mm

**Table 2:** Details of samples prepared for the characterization and evaluation of the CO-NEG.

Formulation	Composition
NEG1	HPC hydrogel loaded with optimized CO-NE
NEG2	HPC hydrogel loaded with NE formulated with oleic acid instead of CO
G3	Aqueous dispersion of CO-NE without HPC gel base
G4	HPC hydrogel (plain)

**Table 3:** The independent factors and responses for the design of experiments.

Run	Factor A	Factor B	Factor C	Response 1		Response 2		Response 3	
	Cinnamon Oil (%)	S_mix_ (%)	HLB	Droplet Size (nm)		Stability Index (%)		Zone of Inhibition (mm)	
				Observed	Predicted	Observed	Predicted	Observed	Predicted
1	15.8	34.818	10	160	163.04	70	69.86	13	12.88
2	20	34.05	12.62	119	117.45	82	83.36	22	22.81
3	20	30	10.22	174	171.95	69	68.12	19	18.51
4	19.12	38.9	12.02	115	117.14	84	83.54	21	20.18
5	19.72	35.1	10.6129	146	147.48	74	73.68	20	20.56
6	15.24	40	12.68	110	108.46	85	87.28	12	12.80
7	20	40	14	89	90.68	96	94.65	24	23.25
8	12	36.15	14	95	97.90	93	91.05	11	10.57
9	20	40	10	140	138.08	74	73.95	20	20.52
10	12	36.5	11.82	134	131.63	78	80.02	10	10.02
11	12	40	10.26	140	141.17	76	74.39	9	8.84
12	17	30	14	100	100.53	88	87.33	16	15.97
13	12	30	12.32	142	140.93	80	78.07	9	8.82
14	16	35	12	125	129.36	79	80.36	15	14.46
15	15.8	34.818	10	161	163.04	70	69.86	13	12.88
16	12	30	10	191	189.24	65	66.07	7	7.57
17	16.153	35.15	11.804	130	132.01	77	79.47	15	14.59
18	16.08	39.75	10.66	137	133.98	79	76.75	14	13.35
19	15.24	40	12.68	111	108.46	86	87.28	12	12.80
20	17	30	14	101	100.53	87	87.33	16	15.97
21	17.16	36.05	14	98	94.97	92	91.57	17	17.66

**Table 4:** Optimum formula suggested for the CO-NE formulation and the predicted and observed responses for the optimized formula.

Independent Factors		
Factor	Name	Value
A	Cinnamon oil Percent	20%
B	S_mix_ percent	40%
C	HLB of S_mix_	14.
Responses	Predicted value	Observed value
Droplet size (nm)	90.7	92.0 ± 3
Stability index (%)	94.6	95.0 ± 2
Zone of inhibition (mm)	23.3	23.0 ± 1.5

**Table 5:** Results of in vivo local anesthetic activity on rats using HPC hydrogel loaded with optimized CO-NE (NEG1), HPC hydrogel loaded with NE formulated with oleic acid instead of CO (NEG2), aqueous dispersion of CO-NE without HPC gel base (G3), and plain HPC hydrogel (G4).

Sample	Latency Reaction Time (sec) Observed after Different Time Intervals				
	5 min	15 min	30 min	45 min	60 min
NEG1	18.5 ± 1.5	23.5 ± 2.5	31.0 ± 2.0	17.5 ± 1.5	8.5 ± 1.5
NEG2	9.5 ± 1.0	10.5 ± 1.5	8.5 ± 1.5	8.0 ± 1.0	8.0 ± 1.0
G3	14.5 ± 1.0	22.0 ± 2.0	19.5 ± 1.0	9.0 ± 1.5	7.5 ± 0.5
G4 (control gel)	8.0 ± 0.5	8.5 ± 0.5	8.0 ± 0.5	7.0 ± 0.5	7.5 ± 1.0

## Data Availability

Not applicable.

## Supplementary Materials

The following are available online at https://www.mdpi.com/article/10.3390/pharmaceutics13071008/s1, Table S1: ANOVA data for globule size of CO-NE formulations in various runs, Table S2: ANOVA data for the stability index of the CO-NE formulations in various runs, Table S3: ANOVA data for the zone of inhibition of the CO-NE formulations in various runs, Table S4: Coefficient Table indicated p-values for each of selected factor on each response.
