# Supplementary material for: Development and Optimization of Cinnamon Oil Nanoemulgel for Enhancement of Solubility and Evaluation of Antibacterial, Antifungal and Analgesic Effects against Oral Microbiota
Source: Pharmaceutics. 2021 Jul 2;13(7):1008. doi: 10.3390/pharmaceutics13071008 (PMC8309164; doi:10.3390/pharmaceutics13071008)
Supplement: Supplementary file 1 [file pharmaceutics-13-01008-s001.zip › pharmaceutics-1237227-supplementary.pdf]

pharmaceutics

Supplementary Materials: Development and Optimization of Cinnamon Oil Nanoemulgel for Enhancement of Solubility and Evaluation of Antibacterial, Antifungal and Analgesic Effects against Oral Microbiota

Khaled M. Hosny, Rasha A. Khallaf, Hani Z. Asfour, Waleed Y. Rizg, Nabil A. Alhakamy, Amal M. Sindi, Hala M. Alkhalidi, Walaa A. Abualsunun, Rana B. Bakhaidar, Alshaimaa M. Almehmady, Wesam H. Abdulaal, Mohammed A. Bakhrebah, Mohammed S. Alsuabeyl, Ahmed K. Kammoun, Adel F. Alghaith and Sultan Alshehri

Table S1. ANOVA data for globule size of CO-NE formulations in various runs.

Source	p-value	Significance
**Model**	< 0.0001	significant
A-Cinnamon Oil percent	0.0006	--
B-S_{mix} percent	< 0.0001	significant
C-HLB	< 0.0001	significant
AB	0.1566	--
AC	0.3663	--
BC	< 0.0001	significant
**Residual**	--	--
Lack of Fit	0.0173	significant

Table S2. ANOVA data for the stability index of the CO-NE formulations in various runs.

Source	p-value	--
**Model**	< 0.0001	significant
A-Cinnamon Oil percent	0.3510	--
B-S_{mix} percent	< 0.0001	significant
C-HLB	< 0.0001	significant
**Residual**	--	--
Lack of Fit	0.0517	not significant

Table S3. ANOVA data for the stability index of the CO-NE formulations in various runs.

Source	p-value	--
**Model**	< 0.0001	significant
A-Cinnamon Oil percent	< 0.0001	significant
B-S_{mix} percent	0.0513	--
C-HLB	0.0002	--
AB	0.4013	--
AC	0.1090	--
BC	0.2107	--
A^2	0.0005	--
B^2	0.0063	--
C^2	0.8512	--
**Residual**	--	--
Lack of Fit	--	--

Table S4. Coeffecient Table indicated p-values for each of selected fator on each re.

	Intercept	A	B	C	AB	AC	BC	A^2	B^2	C^2
**Globule Size**	129.363	−3.78168	−12.8252	−32.6735	1.62239	1.0667	7.90462			
**p-values**		0.0006	< 0.0001	< 0.0001	0.1566	0.3663	< 0.0001			
**Stability Index**	80.3597	0.455694	3.48621	10.3469						
**p-values**		0.3510	< 0.0001	< 0.0001						
**Zone of Inhibition**	14.4553	6.16188	0.487956	1.19226	0.260898	0.554172	-0.383875	1.81754	−1.22267	−0.0738419
**p-values**		< 0.0001	0.0513	0.0002	0.4013	0.1090	0.2107	0.0005	0.0063	0.8512
